# Prospective Audit and Feedback for Antimicrobial Treatment of Patients Receiving Renal Replacement Therapy in Community-Based University Hospitals: A before-and-after Study

**DOI:** 10.3390/ph17070854

**Published:** 2024-06-28

**Authors:** Namgi Park, Jiyeon Bae, Soo Yeon Nam, Ji Yun Bae, Kang-Il Jun, Jeong-Han Kim, Chung-Jong Kim, Kyunghee Kim, Sun Ah Kim, Hee Jung Choi, Sandy Jeong Rhie

**Affiliations:** 1 Department of Pharmacy, Ewha Womans University Mokdong Hospital, Seoul 07985, Republic of Korea; 2 College of Pharmacy and Graduate School of Pharmaceutical Sciences, Ewha Womans University, Seoul 03760, Republic of Korea; 3 Division of Infectious Diseases, Department of Internal Medicine, Ewha Womans University Mokdong Hospital, Seoul 07985, Republic of Koreabaejy@ewha.ac.kr (J.Y.B.); heechoi@ewha.ac.kr (H.J.C.); 4 Division of Infectious Diseases, Department of Internal Medicine, Ewha Womans University Seoul Hospital, Seoul 07804, Republic of Korea; 5 Department of Pharmacy, Ewha Womans University Seoul Hospital, Seoul 07804, Republic of Korea

**Keywords:** antimicrobial stewardship programs, prospective audit and feedback, renal replacement therapy, hemodialysis, continuous renal replacement therapy, mortality

## Abstract

In South Korea, because of manpower and budgetary limitations, antimicrobial stewardship programs have relied on preauthorization. This study analyzed the impact of a prospective audit and feedback (PAF) program targeting inpatients undergoing intermittent hemodialysis or continuous renal replacement therapy, which was implemented at two community-based university hospitals. During three years of PAF, 27,906 antimicrobial prescriptions were reviewed, with 622 (2.2%) interventions. The mean incidence density per 1000 patient days of multidrug-resistant organisms, except for carbapenem-resistant *Acinetobacter baumannii*, decreased in the study population, whereas it increased among inpatients. Multivariable Poisson regression analysis revealed that after PAF, the incidences of vancomycin-resistant *Enterococcus* and mortality decreased (incidence risk ratio, 95% confidence interval: 0.53, 0.31–0.93 and 0.70, 0.55–0.90, respectively). Notably, after PAF, incorrect antimicrobial dosing rates significantly decreased (tau −0.244; *p* = 0.02). However, the incidences of other multidrug-resistant organisms, *Clostridioides difficile*, length of stay, and readmission did not significantly change. This study shows that in patients undergoing intermittent hemodialysis or continuous renal replacement, targeted PAF can significantly reduce multidrug-resistant organism rates and all-cause hospital mortality, despite limited resources. Furthermore, it can improve antimicrobial dosage accuracy.

## 1. Introduction

Antimicrobial stewardship programs (ASPs) are crucial for optimizing antimicrobial use and combating antimicrobial resistance (AMR), which is a growing major threat to global public health [1]. Several studies indicate that in acute care hospital settings, 30–50% of antimicrobial prescriptions may be unnecessary or inappropriate [2], which not only diminishes treatment effectiveness but also increases the risk of adverse events and contributes to AMR development [2]. Inappropriate antimicrobial use has consequences beyond immediate patient outcomes and public health challenges. For instance, Murray et al. estimated that in 2019, up to 4.95 million deaths were attributable to AMR [3]. Moreover, O’Neill et al. projected that AMR-associated deaths may reach 10 million by 2050 [4], underscoring the urgent need for ASP.

The core ASP strategies recommended by the Infectious Diseases Society of America are prospective audit and feedback (PAF) and preauthorization [1]. However, because of their labor-intensiveness, PAF programs are considered hard to introduce [5,6,7], and this implementation challenge is often observed in South Korea, where > 80% of large hospitals heavily rely on preauthorization, and only slightly more than half perform PAF because of limited budgets and a lack of manpower [8,9]. PAF can be more effective than preauthorization, and it offers complementary advantages, including flexible intervention timing and program operation [10,11].

Because patients undergoing renal replacement therapy (RRT) have altered antimicrobial pharmacokinetics, which can affect infection outcomes, optimal antimicrobial doses are very challenging to achieve in these patients because they require personalized adjustments based on a patient’s pharmacokinetic profile, renal function, the pathogen involved, and drug properties [12,13]. Moreover, for continuous renal replacement therapy (CRRT), dosing guidelines are sometimes unavailable, and for critically ill patients, a lack of clinical data about the use of novel antimicrobials during CRRT limits evidence-based dose recommendations [14]. Because these patients frequent healthcare settings, they are at an increased risk of acquiring multidrug-resistant organisms (MDROs). Therefore, patients undergoing RRT may be at a high risk of treatment failure, MDRO infection, and mortality [15]. Although PAF may offer crucial support to patients undergoing RRT, its impact on these patients is yet to be fully investigated.

This study investigated the impact of PAF on microbiological and clinical outcomes in patients undergoing intermittent hemodialysis (IHD) or CRRT, as well as its effects on antimicrobial dosing accuracy in these patients.

## 2. Results

### 2.1. Patient Characteristics

**Figure 1:**
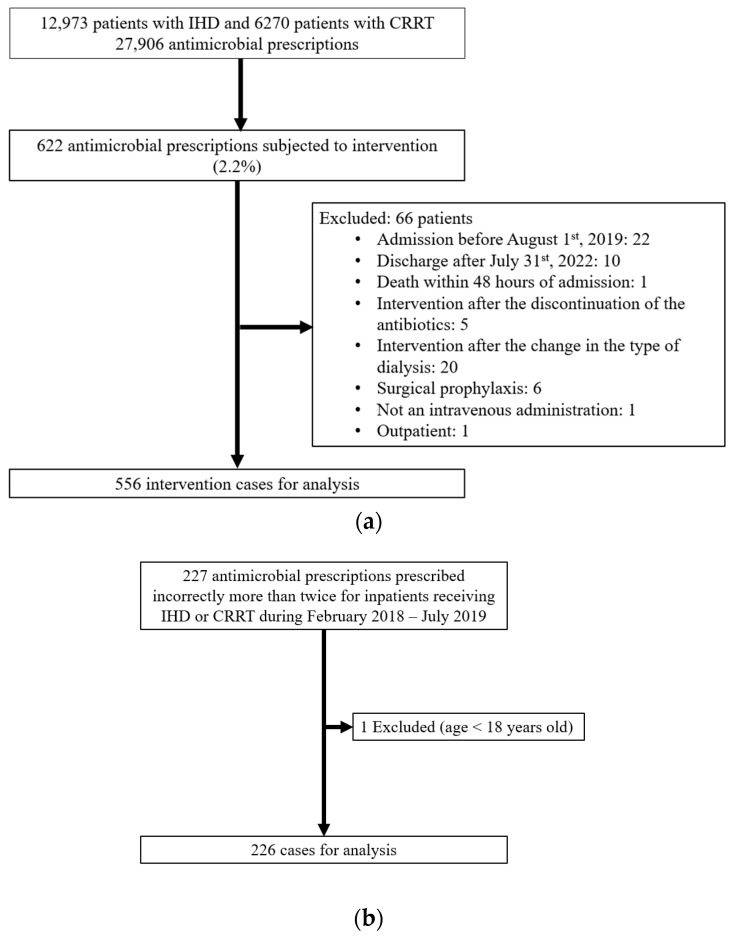
The study flow chart. (**a**) After-PAF. (**b**) Before-PAF. PAF: prospective audit and feedback, IHD: intermittent hemodialysis, and CRRT: continuous renal replacement therapy.

In this before-and-after study, the before-PAF group included inpatients aged ≥18 years who received incorrect antimicrobial prescriptions and underwent IHD or CRRT between February 2018 and July 2019, while the after-PAF group involved adult inpatients who underwent RRT and received PAF antimicrobial prescription intervention between August 2019 and July 2022. During the three-year PAF period, 27,906 antimicrobial prescriptions, involving 12,973 and 6270 patients with IHD and CRRT, respectively, were reviewed. Of these prescriptions, 622 (2.2%) required intervention, and 556 were included in this study (Figure 1). Of these interventions, 280 cases were accepted (acceptance rate: 50.4%). In the before-PAF group, 226 prescriptions were included. 

The demographic and clinical characteristics of both groups are shown in Table 1. The after-PAF group was older than the before-PAF group (mean age: 70.7 vs. 67.4 years; *p* = 0.01). Regarding antimicrobial class, in the before- and after-PAF groups, the incorrect dosing rate was highest for β-lactam/β-lactamase inhibitors (BL/BLI [67/226, 29.6%]) and carbapenems (165/556, 29.7%), respectively. In both groups, a Charlson Comorbidity Index (CCI) score of ≤2 was most prevalent. However, severity was higher in the after-PAF group vs. the before-PAF group (CCI score: ≥3 in 57.7% vs. 40.7% of cases, and ≥5 in 22.8% vs. 8.8% of cases; *p* < 0.01). 

### 2.2. Outcomes

#### 2.2.1. Incidences of Carbapenem-Resistant *Acinetobacter baumannii* (CRAB), Carbapenem-Resistant Enterobacterales (CRE), Methicillin-Resistant *Staphylococcus aureus* (MRSA), and Vancomycin-Resistant *Enterococcus* (VRE)

**Figure 2:**
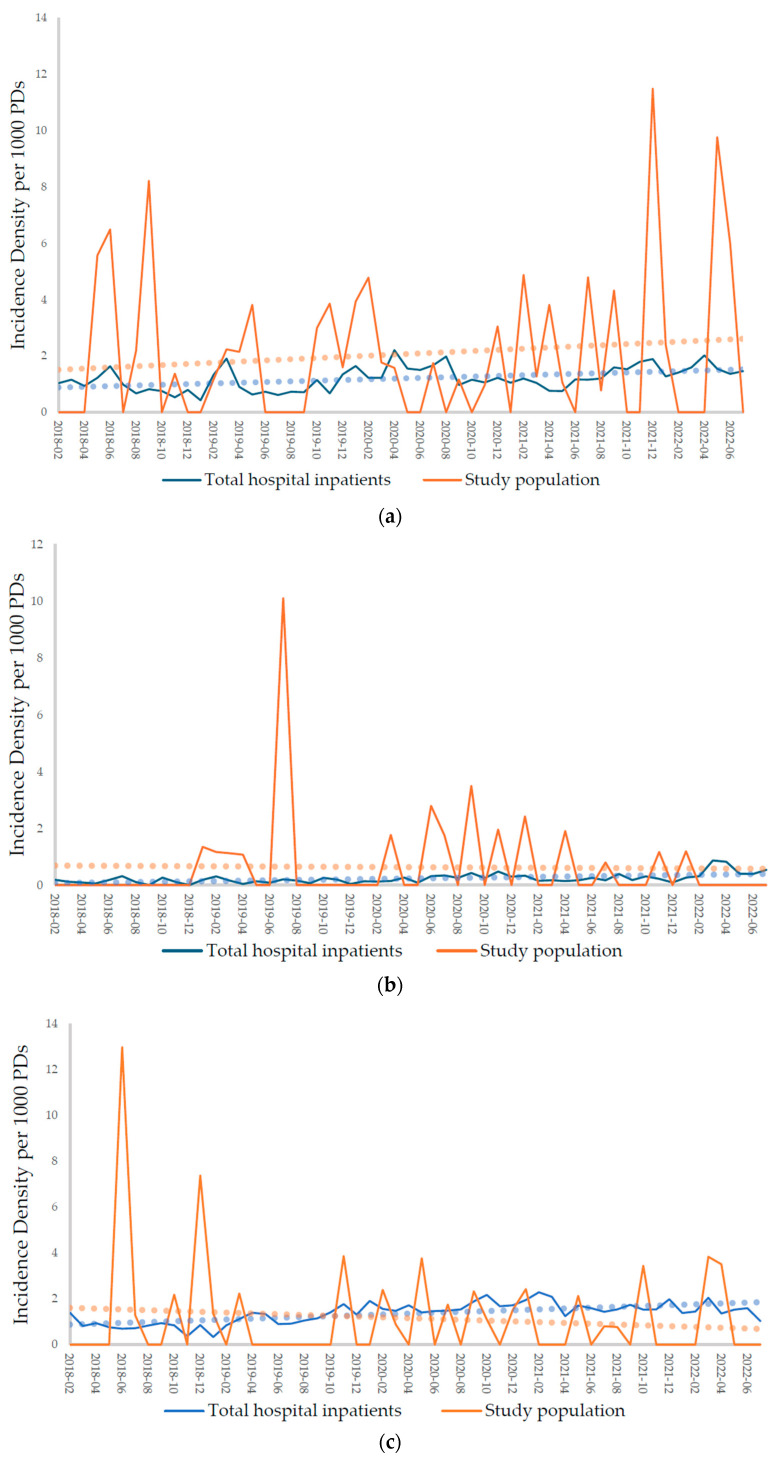

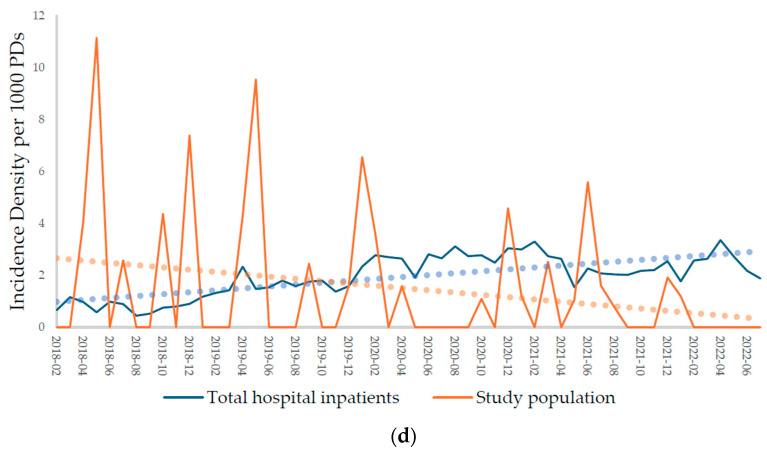
Monthly CRAB, CRE, MRSA, and VRE incidence densities (per 1000 PDs) in the total hospital inpatient and study population groups. The blue and orange dotted lines are the total hospital inpatient and the study populations’ trend lines, respectively. (**a**) CRAB. (**b**) CRE. (**c**) MRSA. (**d**) VRE. PDs: patient days, CRAB: carbapenem-resistant *Acinetobacter baumannii*, CRE: carbapenem-resistant Enterobacterales, MRSA: methicillin-resistant *Staphylococcus aureus*, and VRE: vancomycin-resistant *Enterococcus*.

The study population’s and hospital inpatients’ monthly CRAB, CRE, MRSA, and VRE incidence densities per 1000 patient days (PDs) are shown in Figure 2 (values are shown in Supplementary Table S1). While the monthly MRSA and VRE incidences (per 1000 PDs) decreased over time in the study population, they increased among the total hospital inpatient group. However, CRAB’s monthly incidence increased in the study population. The data were insufficient for temporal trend analysis because there was a high rate of zero values, indicating no occurrence. Table 2 shows the mean incidence densities (per 1000 PDs) before and after the PAF periods. In the total hospital inpatient group, in the before-PAF period, CRAB, CRE, MRSA, and VRE had mean incidences (per 1000 PDs) of 0.95, 0.14, 1.12, and 1.10, respectively, which changed to 1.33, 0.28, 1.64, and 2.38, respectively, after PAF implementation. In the study population, which included patients who underwent IHD or CRRT and were on antimicrobials, in the before-PAF period, CRAB, CRE, MRSA, and VRE had mean incidences (per 1000 PDs) of 1.85, 0.82, 1.34, and 2.41, respectively, which changed to 2.17, 0.53, 1.02, and 1.04, respectively, in the after-PAF period. In the total hospital inpatient group, all mean incidences increased between the before- and after-PAF periods, while in the study population, the mean CRE, MRSA, and VRE, but not CRAB, incidences decreased. 

#### 2.2.2. Comparison of Microbiological and Clinical Outcomes

Next, we compared the microbiological and clinical outcomes using multivariable Poisson regression analysis with adjustments for various confounding variables, including age group, sex, CCI, RRT type, and length of stay (LOS) at baseline (Table 3). This analysis revealed that the VRE incidence rate was significantly associated with PAF interventions (incidence risk ratio: 0.53; 95% confidence interval: 0.31–0.93; *p* = 0.03). However, PAF was not significantly associated with CRAB, CRE, MRSA, or *C. difficile* infection (CDI) incidence rates. Regarding clinical outcomes, all-cause hospital mortality significantly reduced after PAF implementation (incidence risk ratio: 0.70; 95% confidence interval: 0.55–0.90; *p* = 0.01). However, readmission within 30 days and an LOS of > 30 days were not significantly associated with PAF.

### 2.3. Incorrect Antimicrobial Dosing Rate

**Figure 3:**
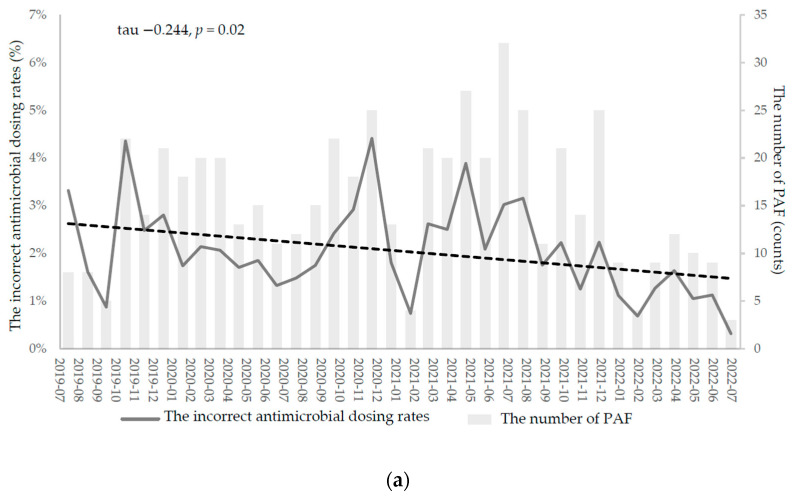

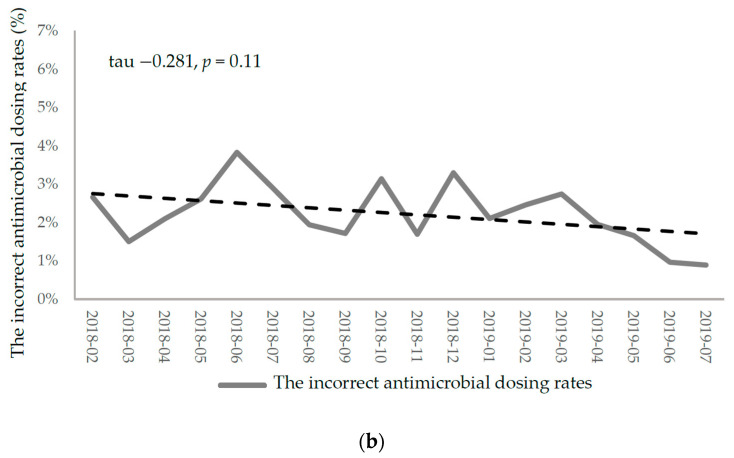
The monthly trend of incorrect antimicrobial dosing rates. (**a**) After-PAF. (**b**) Before-PAF. PAF: prospective audit and feedback.

Incorrect antimicrobial dosing is an antimicrobial prescription dosage that deviates from dosing protocols through overdosing, underdosing, or inappropriate timing. During the pre- and post-PAF periods, the rates of incorrect antimicrobial dosing were 2.14% (226/10,556 cases) and 1.99% (556/27,906 cases), respectively, with most incorrect cases involving the prescription of antimicrobial dosing meant for patients with normal renal function. A modified Mann–Kendall’s test revealed that in the after-PAF period, the monthly rates of incorrect antimicrobial prescription dosing significantly decreased over time (tau −0.244; *p* = 0.02) compared with before-PAF (tau −0.281; *p* = 0.11; Figure 3).

## 3. Discussion

This study shows that implementing a target-specific PAF approach in community-based university hospitals with limited resources improved the outcomes of patients undergoing IHD or CRRT. The VRE incidence and all-cause hospital mortality rates significantly reduced after PAF implementation.

Although a direct comparison of the total hospital inpatient vs. the study population groups was challenging, distinct overall CRE, MRSA, and VRE mean incidence increases were evident during the pre- and post-PAF periods, and decreases were observed in the study population. In South Korea, as part of MDRO surveillance, CRAB, CRE, MRSA, and VRE incidences must be reported to the local health center, whether they occur as infections or colonization. Additionally, for isolation room prioritization, CRE and VRE cases are subject to hospital regulations. Therefore, during the study period, it was possible to collect monthly CRAB, CRE, MRSA, and VRE incidence data in the total hospital inpatient population. Despite some constraints, these data gave insights into the overall hospital MDRO incidence and facilitated its comparison with the PAF study population. The incidence rate (per 1000 PDs) graphs revealed an intersection between MRSA and VRE lines, indicating that PAF influenced the study population. Because our PAF program focused on patients undergoing IHD and CRRT, Gram-positive cocci, including *Staphylococcus aureus*, were the main cause of bloodstream bacterial infections during dialysis [16,17]. It is evident that when compared with CRAB and CRE, our program had a greater impact on MRSA and VRE incidences, which are Gram-positive cocci. However, the trend showed an increase in CRAB, probably because the PAF program had a relatively limited effect on it. Other studies also indicate that ASPs had different impacts on various MDROs [18,19,20,21]. Our study population was made up of patients undergoing RRT, which may have also contributed to the differences in vulnerability. Although the PAF intervention group was statistically limited because of a high rate of zero values in the monthly incidence rate data, notably, it exhibited a contrasting decreasing trend when compared with the total hospital inpatient population. Multivariable Poisson regression analysis indicated that in the after-PAF period, this change was statistically significant.

The before- and after-PAF group comparison revealed a significant decrease in the VRE incidence, which, albeit not directly, is attributable to a PAF-facilitated reduction in incorrect antimicrobial dosing rates. Because vancomycin was not among the antimicrobials covered by the PAF intervention, its dosage was based on vancomycin therapeutic drug monitoring recommendations, regardless of patient dialysis status. However, incorrect non-vancomycin antimicrobial prescriptions may have affected the VRE incidence. Various risk factors, including antibiotic pressure, clinical condition severity, previous MDRO colonization or infection, and colonization pressure, are associated with VRE acquisition [22,23]. Moreover, incorrect antimicrobial prescriptions contribute to antimicrobial resistance [24]. In this study, during the after-PAF period, incorrect antimicrobial dosing rates fell from 2.14% to 1.99% because of the program’s educational effect. Nonetheless, although our study population was small compared with the number of inpatients who received antimicrobial therapy while undergoing IHD or CRRT, this finding remains noteworthy. PAF implementation may have improved guideline adherence by enhancing physician awareness about the regimens recommended for patients undergoing IHD or CRRT. This positive outcome highlights the educational influence PAF has on clinicians and how it enhances ASP visibility in hospital environments [1].

Furthermore, the lack of a significant decrease in the incidence of other MDROs, such as CRAB, CRE, and MRSA, as well as CDI, does not necessarily imply PAF implementation’s ineffectiveness. In this study, significant differences were not observed between CRAB, CRE, MRSA, and CDI incidences, which is consistent with previous findings [18,19,20,21]. The lack of a significant decrease may also indicate no significant increase in MDROs and CDI incidence. 

The significant mortality rate reduction and absence of significant LOS and readmission rates are consistent with previous findings [20,25,26]. Here, we observed a 30% reduction in all-cause hospital mortality (IRR: 0.70; 95% CI: 0.55–0.90). After primary prescribers accepted intervention for inappropriate carbapenem use, Seah et al. observed a notable decrease in mortality [25]. Moreover, there were no significant differences in LOS and 30-day readmission rates, which is attributable to a large proportion of the patients having high acuity and multiple comorbidities [25]. Our study group was made up of patients undergoing RRT and a high proportion of patients in the after-PAF group with CCI scores of ≥3, suggesting the presence of multiple comorbidities and a highly critical status.

However, several factors may have influenced the observed low PAF intervention acceptance rate, which was lower than those reported in other studies [27,28,29]. First, the study population was made up of patients receiving IHD or CRRT, which indicates that they may have had comorbidities or severe conditions that often necessitate polypharmacy. Because of concerns over treatment failure, critically ill patients are frequently subjected to antimicrobial overuse [30]. Additionally, we noted that when interventions were declined, there was a lack of active communication between primary physicians and the PAF team, and reasons for rejection were rarely documented. Furthermore, PAF intervention was confined to cases in which antimicrobial prescription deviated from dosing and dosing time protocols. Therefore, expanding the intervention range and specificity may increase acceptance rates. Moreover, efforts to improve communication and foster collaboration between primary physicians and the PAF team are crucial, and they can be achieved by enhancing pharmacists’ communication skills and supporting PAF service expansion to the entire hospital.

In this study, targeting PAF implementation to patients undergoing IHD or CRRT highlights the importance of offering PAF service regardless of patient cohort size. To combat the rising MDRO incidence, Korean ASP guidelines recommend the selective integration of preauthorization and PAF [31,32,33,34,35]. However, PAF implementation faces several obstacles, including time constraints, personnel shortages, and a lack of compensation [8]. Kim et al. found that in Korea, at 88.1% of hospitals with >500 beds, the main ASP strategy was preauthorization and restriction, with PAF intervention for inappropriate antimicrobial dosage accounting for only 20.2%. Even at Ewha Womans University Medical Center (EUMC), because of staffing shortages, PAF implementation timing was considerably later than that of preauthorization and restriction, with a focus on monitoring antimicrobial dosage in patients undergoing IHD or CRRT. Microbiological and clinical outcome analysis revealed a significant decrease or no significant differences, confirming its benefit and safety. Considering PAF’s numerous advantages, the pursuit of add-on PAF is crucial, regardless of the patient cohort size or the number of interventions adopted. Hospitals are encouraged to choose the PAF intervention methods that best suit their needs, including antimicrobial dosing management, broad-spectrum antimicrobial de-escalation, and replacing intravenous medications with oral ones. The target patient population can then be gradually expanded as resources allow.

This study had some limitations. First, its retrospective nature and time effect may have introduced bias. Second, the patients who received intervention and were included in the analysis were a fraction of the monitored patients with IHD or CRRT, who received antimicrobial therapy. Furthermore, the decision to target the PAF program to a specific group of patients was based on the practical constraints imposed by limited resources. Nonetheless, the significant decrease in VRE incidence and mortality, as well as the reduction in the rate of incorrect antimicrobial prescription dosing, highlight PAF’s benefits. Thirdly, this study did not cover all MDROs, and extended-spectrum beta-lactamase-producing bacteria and carbapenem-resistant *Pseudomonas aeruginosa* were not included. Instead, we focused on CRAB, CRE, MRSA, and VRE incidence rates (per 1000 PDs) since these MDROs pose notable challenges in our hospitals and warrant thorough investigation. Furthermore, pharmacoeconomic outcomes like the defined daily dose could not be calculated. Data on each antimicrobial’s overall use would describe each MDRO’s incidence better. Finally, this study did not distinguish true infections from colonization, and infection source information was also missing. Further investigations that determine how PAF implementation affects other antimicrobial-resistant bacteria, including by verifying infection status, are needed. 

## 4. Materials and Methods

### 4.1. PAF Implementation

PAF was implemented at EUMC, a community-based tertiary medical center made up of a 700-bed (EUMC Mokdong Hospital) and a 747-bed (EUMC Seoul Hospital) hospital. The general aspects and patient characteristics of these hospitals are similar to those of other tertiary hospitals. PAF initiation was led by a leadership group made up of infectious disease (ID) faculty specialists, a faculty member from the School of Pharmacy of Ewha Womans University, and the pharmacy departments’ directors. To maximize operational efficiency with limited resources, PAF was targeted to patients undergoing IHD or CRRT. The dosing protocol development was based on the RRT type and referred to guidelines from the Korean Society for Chemotherapy (http://ksc.thepowerbrains.com/search/main.do (accessed on 1 July 2019)) [36] and the Sanford Guide to Antimicrobial Therapy and Micromedex (Truven Health Analytics). The protocol included dosing recommendations for penicillin, cephalosporins, carbapenems, BL/BLIs, quinolones, metronidazole, and colistin. To ensure accessibility to all healthcare professionals at the hospitals, through collaboration with the information technology department of EUMC, the dosing protocols were embedded into the electronic medical record system. Based on the dosing protocols, PAF manuals with instructions on how to access the list of patients undergoing antimicrobial therapy during IHD or CRRT, evaluate prescriptions based on dialysis status, implement interventions as needed, and document intervention outcomes were developed for pharmacists. All antimicrobial prescriptions of patients undergoing IHD or CRRT were reviewed by pharmacists. Prescribed antimicrobial dosage deviations from dosing protocol guidelines because of overdosing, underdosing, or inappropriate timing were considered inappropriate or incorrect antimicrobial dosing, and this was followed by recommendations. To encourage pharmacist interventions, the PAF service was provided at least thrice weekly using electronic co-signatures from ID specialists. On a part-time basis, a pharmacist and an ID specialist were involved in the PAF service at each hospital.

#### MDRO Screening Programs at the Hospitals

EUMC’s intensive care unit and general ward have distinct MDRO screening programs. For all patients admitted into the intensive care unit, rectal swabs and nasal/transthoracic aspiration samples are taken for initial CRE and CRAB screening, respectively, followed by weekly surveillance. Although VRE screening was not conducted, patients whose clinical specimens tested VRE-positive through any microbiological tests underwent weekly monitoring via rectal swab VRE tests. General ward patients were exempt from CRE, CRAB, and VRE screening unless they had colonization. The diagnosis of extended-spectrum beta-lactamase-producing Enterobacteriaceae, carbapenem-resistant *Pseudomonas aeruginosa*, and MRSA primarily depended on each patient’s antimicrobial susceptibility results. Notably, separate screening tests were not available for these MDROs. Because of hospital policy and limited room availability, patients with VRE or CRE were prioritized for isolation.

### 4.2. Study Design

This before-and-after study was conducted after three years of PAF service. This study involved inpatients aged ≥ 18 years, who were undergoing IHD or CRRT and were undergoing antimicrobial therapy. A before-and-after design was used because of ethical considerations since the inclusion of control groups risked suboptimal antimicrobial treatment. The before- and after-PAF groups included cases of incorrect antimicrobial prescription among patients hospitalized between February 2018 and July 2019 and patients hospitalized between August 2019 and July 2022 who had received PAF intervention antimicrobial prescriptions, respectively. Compared with the after-PAF period, the before-PAF period was shorter because of the hospital software systems transition on 1 February 2018, which made data retrieval for the period before the transition challenging, thereby limiting the available data and restricting the control period to 18 months. In the before-PAF group, antimicrobial prescription doses were reviewed, and those that deviated from the dosing protocols on more than two occasions were selected. This action was based on the understanding that physicians may prescribe incorrect doses at antimicrobial therapy initiation, but this happening more than twice may indicate a pattern of incorrect dosing. If patients were simultaneously prescribed multiple antimicrobials, each was considered an independent prescription.

Patients hospitalized before the study period, those discharged after the study period had ended, those who died within 48 h of admission, those who used antimicrobials as surgical prophylaxis, or those treated with non-systemic antimicrobials were excluded from this study. In the after-PAF group, those who discontinued antimicrobials at the time of intervention or changed the RRT type before the intervention were excluded.

### 4.3. Data Collection

Demographic, CCI score, RRT type, antimicrobial prescription, microbiology culture, sensitivity report, *Clostridioides difficile* toxin, date of hospital death (if it occurred), and admission date data were obtained from the electronic medical record system. In the after-PAF group, data on the number and type of interventions, as well as intervention acceptance, were also obtained.

The microbiological outcomes of interest were CRAB, CRE, MRSA, and VRE colonization, as well as CDI incidence rates. Culture and sensitivity tests on patient samples were used to identify positive CRAB, CRE, MRSA, and VRE cases. CDI status was based on a positive *C. difficile* toxin gene result or *C. difficile* toxin A&B assays.

All-cause hospital mortality, an LOS of >30 days, and readmission within 30 days of discharge were the clinical outcomes of interest. Incorrect antimicrobial dosing rates were used to assess changes in antimicrobial dosing accuracy in patients undergoing IHD or CRRT.

### 4.4. Statistical Analyses

Categorical and continuous variables were compared using a chi-squared test and Student’s *t*-test, respectively. For the study period, monthly CRAB, CRE, MRSA, and VRE incidence rates (per 1000 PDs) were compared with the monthly incidence rates of all hospital inpatients, which were regularly collected by hospital infection control units. Because the number of zero values (no incidence) posed significant statistical limitations when assessing temporal trends, multivariable Poisson regression analysis was used to compare the microbiological and clinical outcomes of interest in the before- and after-PAF groups, with adjustment for several confounders, including age group, sex, CCI, RRT type, and LOS at baseline [37,38,39,40,41,42]. A modified Mann–Kendall’s trend test designed for serially correlated data [43] was used to analyze incorrect antimicrobial dosing rates. *p* < 0.05 indicated statistically significant differences. All statistical analyses were performed using R 4.3.3 software (R Core Team, Vienna, Austria).

## 5. Conclusions

ASP implementation for inpatients undergoing RRT has not been fully investigated because of individualized antimicrobial dosing complexities and a scarcity of CRRT patient dosing guidelines. Here, we developed in-hospital dosing protocols, introduced a PAF service, and assessed their effects on microbiological and clinical outcomes. Our analysis revealed that VRE incidence, mortality, and incorrect antimicrobial dosing rates were significantly improved. Our findings highlight the feasibility and reliability of PAF service implementation, even in resource-limited hospital settings. For optimized antimicrobial use and improved patient outcomes, we propose widespread PAF use in patients undergoing RRT, regardless of resource constraints. Future studies should investigate the potential benefits of tailoring PAF interventions to patients undergoing RRT.

## Figures and Tables

**Table 1:** Patient characteristics.

Characteristics	Before-PAF (*n* = 226)	After-PAF (*n* = 556)	*p*
Mean age: years (SD)	67.4 (12.6)	70.7 (12.5)	0.01
Age group: *n* (%)			<0.01
≤39	8 (3.5)	4 (0.7)	
40–49	10 (4.4)	37 (6.7)	
50–59	41 (18.1)	61 (11.0)	
60–69	58 (25.7)	132 (23.7)	
70–79	75 (33.2)	174 (31.3)	
80–89	26 (11.5)	127 (22.8)	
≥90	8 (3.5)	21 (3.8)	
Sex: *n* (%)			0.81
Male	138 (61.1)	345 (62.1)	
Female	88 (38.9)	211 (37.9)	
Type of RRT: *n* (%)			0.21
IHD	170 (75.2)	392 (70.5)	
CRRT	56 (24.8)	164 (29.5)	
Antibiotic class: *n* (%)			<0.01
Penicillin	1 (0.4)	2 (0.4)	
Cephalosporins			
1st CEPs	8 (3.5)	42 (7.6)	
2nd CEPs	4 (1.8)	10 (1.8)	
3rd CEPs	12 (5.3)	20 (3.6)	
4th CEPs	25 (11.1)	23 (4.1)	
Carbapenems	37 (16.4)	165 (29.7)	
BL/BLIs	67 (29.6)	140 (25.2)	
Quinolones	19 (8.4)	70 (12.6)	
Nitroimidazole			
Metronidazole	11 (4.9)	11 (2.0)	
Polymixins			
Colistin	42 (18.6)	73 (13.1)	
CCI: *n* (%)			<0.01
≤2	134 (59.3)	235 (42.3)	
3 or 4	72 (31.9)	194 (34.9)	
≥5	20 (8.8)	127 (22.8)	
Previous admission within 30 days: *n* (%)			0.56
N	176 (77.9)	443 (79.7)	
Y	50 (22.1)	113 (20.3)	

PAF: prospective audit and feedback, SD: standard deviation, RRT: renal replacement therapy, IHD: intermittent hemodialysis, CRRT: continuous renal replacement therapy, BL/BLIs: β-lactam/β-lactamase inhibitors, CEPs: cephalosporins, and CCI: Charlson Comorbidity Index.

**Table 2:** Mean CRAB, CRE, MRSA, and VRE incidence densities (per 1000 PDs) in the total hospital inpatient group and the study population group. PAF: prospective audit and feedback, SD: standard deviation, CRAB: carbapenem-resistant *Acinetobacter baumannii*, CRE: carbapenem-resistant Enterobacterales, MRSA: methicillin-resistant *Staphylococcus aureus*, and VRE: vancomycin-resistant *Enterococcus*.

Mean (SD)	Total Hospital Inpatients		Study Population	
	Before-PAF	After-PAF	Before-PAF	After-PAF
CRAB	0.95 (0.37)	1.33 (0.38)	1.85 (2.49)	2.17 (2.73)
CRE	0.14 (0.09)	0.28 (0.18)	0.82 (2.30)	0.53 (0.95)
MRSA	1.12 (0.40)	1.64 (0.28)	1.34 (2.76)	1.02 (1.34)
VRE	1.10 (0.47)	2.38 (0.51)	2.41 (3.54)	1.04 (1.66)

**Table 3:** Multivariable Poisson regression analysis of microbiological and clinical outcomes.

Outcome ^1^	Crude IRR (95% CI)	*p*	Adjusted IRR (95% CI)	*p*
Microbiological				
CRAB	1.15 (0.66–2.00)	0.63	0.99 (0.56–1.76)	0.99
CRE	1.30 (0.48–3.55)	0.61	1.38 (0.50–3.87)	0.54
MRSA	0.56 (0.28–1.09)	0.09	0.56 (0.29–1.06)	0.08
VRE	0.48 (0.29–0.82)	<0.01	0.53 (0.31–0.93)	0.03
CDI	1.29 (0.67–2.46)	0.45	1.49 (0.76–2.92)	0.24
Clinical				
All-cause hospital mortality	0.77 (0.61–0.98)	0.03	0.70 (0.55–0.90)	0.01
Readmission within 30 days	1.33 (0.83–2.14)	0.24	1.25 (0.76–2.05)	0.24
LOS longer than 30 days	0.88 (0.71–1.09)	0.25	0.88 (0.70–1.09)	0.38

^1^ Adjusted variables: age group, sex, Charlson Comorbidity Index, RRT type, and length of stay until incorrect antimicrobial dosing. IRR: incidence risk ratio, CI: confidence interval, CRAB: carbapenem-resistant *Acinetobacter baumannii*, CRE: carbapenem-resistant Enterobacterales, MRSA: methicillin-resistant *Staphylococcus aureus*, VRE: vancomycin-resistant *Enterococcus*, CDI: *Clostridioides difficile* infection, and LOS: length of stay.

## Data Availability

The data presented in this study are available upon request from the corresponding author. The data are not publicly available due to privacy or ethical restrictions.

## Supplementary Materials

The following supporting information can be downloaded at https://www.mdpi.com/article/10.3390/ph17070854/s1. Table S1: The monthly incidence densities of CRAB, CRE, and VRE per 1000 patient days in the study population and the total hospital inpatients.
