# Supplementary material for: Prospective Audit and Feedback for Antimicrobial Treatment of Patients Receiving Renal Replacement Therapy in Community-Based University Hospitals: A before-and-after Study
Source: Pharmaceuticals (Basel). 2024 Jun 28;17(7):854. doi: 10.3390/ph17070854 (PMC11279571; doi:10.3390/ph17070854)
Supplement: Supplementary file 1 [file pharmaceuticals-17-00854-s001.zip › pharmaceuticals-3015912-supplementary.pdf]

Table S1 The monthly incidence densities of CRAB, CRE, MRSA, VRE per 1000 patient days in the study population and the total hospital inpatients

Months	Study population				Total hospital inpatients			
	CRAB	CRE	MRSA	VRE	CRAB	CRE	MRSA	VRE
Feb.2018	0.000	0.000	1.394	0.000	1.030	0.182	0.000	0.667
Mar.2018	0.000	0.000	0.813	0.000	1.161	0.116	0.000	1.161
Apr.2018	0.000	0.000	0.936	4.049	0.936	0.089	0.000	0.981
May.2018	5.571	0.000	0.758	11.142	1.224	0.058	0.000	0.583
Jun.2018	6.494	0.000	0.699	0.000	1.631	0.175	12.987	0.990
Jul.2018	0.000	0.000	0.715	2.577	0.983	0.313	1.289	0.893
Aug.2018	2.198	0.000	0.837	0.000	0.669	0.112	0.000	0.446
Sep.2018	8.219	0.000	0.940	0.000	0.822	0.000	0.000	0.528
Oct.2018	0.000	0.000	0.842	4.367	0.753	0.266	2.183	0.753
Nov.2018	1.370	0.000	0.372	0.000	0.531	0.106	0.000	0.797
Dec.2018	0.000	0.000	0.849	7.386	0.796	0.000	7.386	0.902
Jan.2019	0.000	1.346	0.331	0.000	0.425	0.189	1.346	1.182
Feb.2019	1.167	1.167	0.847	0.000	1.331	0.302	0.000	1.331
Mar.2019	2.237	1.119	1.112	0.000	1.906	0.159	2.237	1.429
Apr.2019	2.144	1.072	1.393	4.287	0.904	0.038	0.000	2.335
May.2019	3.817	0.000	1.342	9.542	0.626	0.134	0.000	1.476
Jun.2019	0.000	0.000	0.900	0.000	0.728	0.086	0.000	1.543
Jul.2019	0.000	10.101	0.915	0.000	0.610	0.203	0.000	1.796
Aug.2019	0.000	0.000	1.055	0.000	0.731	0.162	0.000	1.583
Sep.2019	0.000	0.000	1.151	2.451	0.711	0.068	0.000	1.761
Oct.2019	2.994	0.000	1.409	0.000	1.152	0.256	0.000	1.793
Nov.2019	3.861	0.000	1.766	0.000	0.667	0.196	3.861	1.374
Dec.2019	1.592	0.000	1.309	1.592	1.348	0.040	0.000	1.586
Jan.2020	3.932	0.000	1.907	6.553	1.640	0.134	0.000	2.342
Feb.2020	4.779	0.000	1.556	3.584	1.220	0.126	2.389	2.776
Mar.2020	1.767	1.767	1.476	0.000	1.224	0.144	0.883	2.701
Apr.2020	1.577	0.000	1.721	1.577	2.206	0.265	0.000	2.648
May.2020	0.000	0.000	1.397	0.000	1.556	0.080	3.766	1.915
Jun.2020	0.000	2.789	1.468	0.000	1.499	0.312	0.000	2.811
Jul.2020	1.748	1.748	1.481	0.000	1.671	0.342	1.748	2.658
Aug.2020	0.000	0.000	1.540	0.000	1.980	0.251	0.000	3.112
Sep.2020	1.164	3.492	1.891	0.000	0.965	0.425	2.328	2.741
Oct.2020	0.000	0.000	2.175	1.096	1.158	0.246	1.096	2.771
Nov.2020	0.978	1.955	1.674	0.000	1.060	0.474	0.000	2.484
Dec.2020	3.053	0.000	1.715	4.580	1.225	0.294	1.527	3.038
Jan.2021	0.000	2.421	1.944	1.211	1.047	0.329	2.421	2.991
Feb.2021	4.878	0.000	2.286	0.000	1.199	0.150	0.000	3.298
Mar.2021	1.261	0.000	2.091	2.522	1.045	0.170	0.000	2.741
Apr.2021	3.817	1.908	1.247	0.000	0.762	0.139	0.000	2.633
May.2021	1.062	0.000	1.705	1.062	0.750	0.171	2.123	1.535
Jun.2021	0.000	0.000	1.590	5.587	1.164	0.256	0.000	2.271
Jul.2021	4.796	0.799	1.431	1.599	1.158	0.170	0.799	2.078

Aug.2021	0.769	0.000	1.535	0.769	1.200	0.391	0.769	2.038
Sep.2021	4.329	0.000	1.738	0.000	1.597	0.177	0.000	2.022
Oct.2021	0.000	0.000	1.529	0.000	1.529	0.306	3.442	2.175
Nov.2021	0.000	1.167	1.540	0.000	1.787	0.220	0.000	2.199
Dec.2021	11.494	0.000	1.981	1.916	1.887	0.094	0.000	2.548
Jan.2022	2.389	1.195	1.376	1.195	1.270	0.265	0.000	1.773
Feb.2022	0.000	0.000	1.444	0.000	1.415	0.318	0.000	2.570
Mar.2022	0.000	0.000	2.048	0.000	1.597	0.868	3.831	2.639
Apr.2022	0.000	0.000	1.360	0.000	2.025	0.822	3.509	3.353
May.2022	9.772	0.000	1.523	0.000	1.547	0.399	0.000	2.721
Jun.2022	5.988	0.000	1.591	0.000	1.364	0.390	0.000	2.176
Jul.2022	0.000	0.000	1.019	0.000	1.465	0.542	0.000	1.880

CRAB: carbapenem-resistant *Acinetobacter baumannii*, CRE: carbapenem-resistant Enterobacterales, MRSA: methicillin-resistant *Staphylococcus aureus*, and VRE: vancomycin-resistant *Enterococcus*.
